# Self‐reported pelvic floor dysfunction 12 months after an obstetric anal sphincter injury in relation to maternal body mass index

**DOI:** 10.1111/aogs.70171

**Published:** 2026-03-05

**Authors:** Linda Hjertberg, Eva Uustal, Marie Blomberg, Sofia Pihl

**Affiliations:** ^1^ Department of Obstetrics and Gynecology Linköping University Norrköping Sweden; ^2^ Department of Biomedical and Clinical Sciences Linköping University Linköping Sweden; ^3^ Department of Obstetrics and Gynecology Linköping University Linköping Sweden

**Keywords:** anal incontinence, body mass index (BMI), dyspareunia, obstetric anal sphincter injury (OASI) patient‐reported outcome (PROM), urinary incontinence

## Abstract

**Introduction:**

The aim of this study was to evaluate self‐reported pelvic floor symptoms 12 months postpartum, in women after a first‐time vaginal delivery complicated by an obstetric anal sphincter injury, according to body mass index. The hypothesis was that dyspareunia, urinary incontinence, and anal incontinence would be more common in overweight and obese women compared with normal weight women 12 months postpartum.

**Material and Methods:**

This is a population‐based cohort study. Altogether, 6595 primiparous women with a first‐time vaginal delivery complicated by an obstetric anal sphincter injury in Sweden between 2014 and 2019 were included in the Swedish Perineal Laceration Registry. Self‐reported pre‐pregnancy data and data from the 12‐month follow‐up questionnaire were retrieved from the Perineal Laceration Registry. Uni‐ and multivariate analyses were used to compare normal weight (BMI ≤ 24.9), overweight (25.0–29.9), and obese (≥30) women in regard to the self‐reported prevalence of dyspareunia, urinary incontinence, and anal incontinence 12 months postpartum.

**Results:**

Overweight and obese women had a decreased risk of dyspareunia compared with normal weight women, aOR 0.82 (CI = 0.68–0.98) and aOR 0.71 (CI = 0.54–0.93), respectively. The absolute rate of dyspareunia was 41% among normal weight women, 38% among overweight women, and 33% among the obese group. In the univariate analyses, episiotomy and the grade of anal sphincter injury did not affect the risk of dyspareunia. There was an increased risk of urinary incontinence among overweight (aOR = 1.51, CI = 1.11–2.04) and obese women (aOR = 2.82, CI = 1.94–4.12) compared with normal weight women. The risk of anal incontinence did not differ between the BMI groups.

**Conclusions:**

Dyspareunia 12 months after an obstetric anal sphincter injury was less common among overweight and obese women than in normal‐weight women. Self‐reported anal incontinence one year after an obstetric anal sphincter injury was equally distributed over the BMI groups 12 months after delivery, which is a new finding and clinically relevant.

## Graphical abstract

Self‐reported dyspareunia one year after an obstetric anal sphincter injury (OASI) is less common among overweight and obese women than in normal weight. The risk for anal incontinence does not differ between the BMI groups one year after an OASI, while the risk for UI increases with increasing BMI.

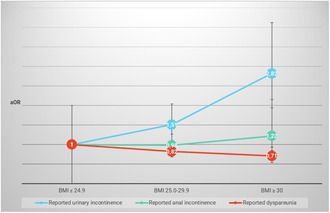

**Abbreviations**
AI: anal incontinence
BMI: body mass index
OASI: obstetric anal sphincter injury
PFD: pelvic floor dysfunction
PLR: Swedish Perineal Laceration Registry
SUI: stress urinary incontinence
UI: urinary incontinence
UUI: urgency urinary incontinence

**Key message**

Dyspareunia 12 months after an OASI is less frequent among overweight and obese women than among normal‐weight women. Self‐reported symptoms of AI do not differ between the BMI groups, while the risk of UI increased with higher BMI.

## 1 INTRODUCTION

Obesity is a global epidemic affecting about one billion (13%) of the adult global population, causing a burden of disease and shorter life expectancy.^1^ The World Health Organization (WHO) defines overweight as a body mass index (BMI) ≥25–29.9, obesity as ≥30–34.9, and morbid obesity as ≥35.^2^

Overweight and obesity during pregnancy are on the rise, with 44.9% of pregnant women in Sweden reaching a BMI over 25, and 16.8% having a BMI ≥ 30.^3^ Obesity during pregnancy is associated with negative maternal and fetal outcomes.

Obese women in general seem to have a higher prevalence of sexual dysfunction, AI and UI compared with normal‐weight women.^4^

The prevalence of obstetric anal sphincter injuries (OASI) among primiparous women in Sweden was 4.6% in 2021.^3^ Risk factors for OASI include primiparity, instrumental delivery, macrosomia, and prolonged duration of the second stage of labor.^5^ These factors can be affected by maternal BMI.^6^, ^7^, ^8^

OASI is a major risk factor for pelvic floor dysfunction (PFD) such as urinary incontinence (UI), pelvic organ prolapse (POP),^9^, ^10^, ^11^ anal incontinence (AI) defined as involuntary loss of feces or gas, and sexual dysfunction, leading to a reduced quality of life.^12^, ^13^ Sexual dysfunction is a broad entity that is influenced by social, physiological, and psychological parameters.^14^ An important parameter of sexual dysfunction is painful intercourse, known as dyspareunia. The prevalence of dyspareunia increases after a labor with a higher degree of perineal tear,^11^ and accordingly, OASI is a well‐known risk factor for dyspareunia at 12 months postpartum.^15^

The role of BMI on the prevalence of dyspareunia among women after OASI has not been previously established. Obese women in general seem to have a higher prevalence of sexual dysfunction, AI and UI than normal‐weight women, independent of perineal tears.^4^ The fact that PFD symptoms are more common among overweight and obese women is commonly attributed to their weight.^16^ However, two previous studies have shown a lower risk for reported AI at eight weeks postpartum among overweight and obese women compared with normal‐weight women.^17^, ^18^ This suggests that it could be of value to clarify the relationship between BMI and PFD after childbirth with an OASI.

The aim of this study was to assess the prevalence of self‐reported pelvic floor dysfunction such as symptoms of dyspareunia, UI, and AI among obese and overweight women compared with normal‐weight women 12 months after a first‐time delivery complicated by an OASI.

We hypothesized that self‐reported symptoms of dyspareunia, AI, and UI would be more common among obese and overweight women than among normal‐weight women 12 months after a first‐time delivery complicated by an OASI.

## 2 MATERIAL AND METHODS

**FIGURE 1:**
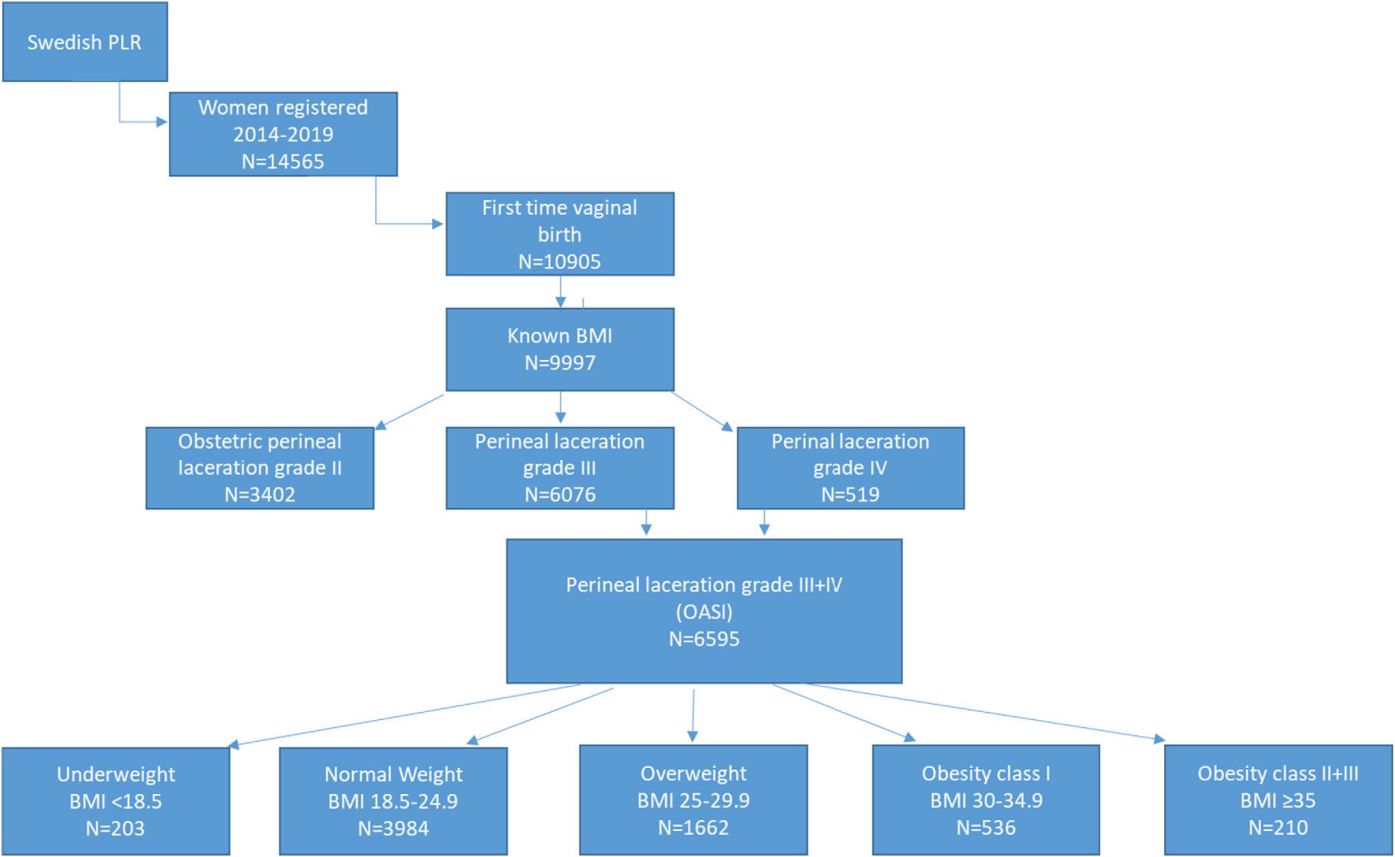
Flowchart of the study population.

This nationwide population‐based cohort study includes 6595 women with a first‐time vaginal birth complicated by an OASI and for whom BMI data were available (Figure 1). Data were retrieved from the Swedish Perineal Laceration Register (PLR) between January 2014 and April 2019. The PLR is a nationwide register that started in 2014 as a section of the Swedish National Quality Register of Gynecological Surgery.^19^ The PLR has previously been described in detail.^20^, ^21^ The Swedish delivery units have joined the register successively since the PLR started, as of 2022 encompasses all Swedish delivery units. The Swedish National Quality Register of Gynecological Surgery, as well as the PLR, provides survey data on patients' self‐reported outcomes and experiences, as well as data extracted from the patient's medical records, and has been validated, demonstrating the validity and reliability of the recorded variable.^22^ The self‐reported data are collected by postal or electronic questionnaires at three points after delivery: data concerning pre‐pregnancy information is collected before discharge from hospital after childbirth, at eight weeks postpartum and at 12 months postpartum, in concerning pelvic floor function and complications.

For the purpose of the present study, the pre‐pregnancy and 12‐month questionnaires were analyzed (Supplemental information Appendices S1 and S2). Questions in the questionnaires refer to several aspects of pelvic floor dysfunction.

Extracted maternal and obstetrical characteristics were age, early pregnancy BMI, the prevalence of diabetes mellitus type I and II, the prevalence of inflammatory bowel disease (Crohn's disease or ulcerative colitis), the prevalence of pre‐pregnancy UI, pre‐pregnancy stress urinary incontinence (SUI) and urgency urinary incontinence (UUI), pre‐pregnancy AI, pre‐pregnancy dyspareunia, pre‐pregnancy sense of bulging of the vagina, pre‐pregnancy sense of wide or tight vagina, fetal presentation at birth, fetal birthweight, duration of second stage of labor, episiotomy at delivery, and mode of vaginal delivery.

Data related to the extent of the perineal laceration and the repair of the perineal laceration concerned: anesthesia during the repair, prophylactic antibiotics during the repair, and suture techniques used for the external sphincter. The study population was divided into five BMI groups: underweight (>18.5 kg/m^2^), normal weight (18.5–24.9 kg/m^2^), overweight (25.0–29.9 kg/m^2^), obesity (30–34.9 kg/m^2^), and morbid obesity (≥35 kg/m^2^) for analysis concerning maternal and obstetrical characteristics as well as repair of the perineal laceration.

For analyses of self‐reported outcomes 12 months postpartum in relation to BMI, the underweight and morbidly obese groups were combined with adjacent categories, resulting in three BMI groups (BMI ≤ 24.9, overweight 25.0–29.9, and obesity ≥30.0 kg/m^2^), as the numbers in the extreme BMI categories were too small for separate analyses. Self‐reported symptoms 12 months postpartum were: the prevalence of overall UI, defined as urinary incontinence once a week or more often, SUI defined as urinary incontinence at physical activity once a week or more often, UUI defined as an urge to void once a week or more often, having had intercourse during the three last months “yes/no,” dyspareunia defined as pain during intercourse “yes/no,” where “yes” included little/moderate/severe/excruciating pain, a sense of a wide vagina during intercourse “yes/no,” a sense of a tight vagina during intercourse “yes/no.” Symptoms suggesting vaginal prolapse were a sense of bulging from the vagina defined as reported once a month or more often, problems with emptying the bowel once a month or more often, a need to press against the posterior vaginal wall, described as “splinting,” during defecation, once a month or more often. Anal incontinence was defined as any reported involuntary loss of feces or flatus.^13^ The Wexner score was calculated based on the supplementary questions, exclusively for women who reported gas and/or fecal incontinence at 12 months postpartum. These participants completed supplementary items assessing incontinence of gas, liquid stool, and solid stool, as well as pad use and lifestyle modifications. Symptom frequency was reported using five predefined categories (never, rarely, 1–3 times per month, 1–3 times per week, daily) to summarize the Wexner score. For this study, we prespecified that symptoms occurring at least once per week represent a clinically meaningful frequency with the potential to influence daily functioning. For dichotomization, women who reported symptoms occurring once weekly or more often were categorized into one group, classified as having clinically relevant symptoms. Women who reported no gas and/or fecal incontinence, and women with symptoms less frequent than once a week were categorized into a comparative group. Women who completed the Wexner score questionnaires were further dichotomized into “Wexner score ≥2” and “Wexner score < 2”. The Wexner score was compared between the BMI groups, with a cut‐off level ≥2 to define a degree of AI that affects the quality of life after an OASI.^10^

### 2.1 Statistical analyses

Data were analyzed using SPSS version 27 (IBM Inc., Armonk, NY). The descriptive analyses of the categorical background data were presented as the total number of patients (n) and percentage of each variable in each BMI group. Continuous data were presented as mean and one standard deviation (SD) or median and interquartile range (IQR), if not normally distributed. Pearson's chi‐square test was used to compare categorical variables. Analysis of variance was used to compare the mean value of age. Nonparametric tests were used to analyze the duration of the second stage of labor in the numerical background data. A *p* value <0.05 was considered significant. Variables with few available cases were not statistically analyzed. Binary logistic regression analyses were used for comparison between three BMI groups (BMI ≤ 24.9, overweight [25.0–29.9], and obesity [≥30]) with BMI ≤ 24.9 set as the reference group. Risk estimates were presented as crude odds ratios (ORs) with 95% confidence intervals (CI). Potential confounders were selected by univariate analysis. Furthermore, a significant relation to the outcome in the primary multivariate analysis was demanded to be included in the final adjusted odds ratio. Multivariate analyses were used for comparison between the BMI groups. Risk estimates are presented as adjusted odds ratios (aORs) with 95% CI.

## 3 RESULTS

The study population consisted of 6595 women with a first‐time vaginal birth complicated by an OASI, data retrieved from the Swedish PLR between 2014 and 2019. In the study population, the prevalence of underweight women was 3.1%, 60.4% were normal weight, 25.2% overweight, and 11.3% obese (8.1% obesity class I and 3.2% total in obesity class II and III).

Obstetric practice characteristics differed according to women's BMI class. There was a significant difference in the usage of episiotomy between the underweight group (17.2%) and the morbidly obese group (8.1%) (*p* < 0.050). The duration of the second stage of labor decreased with higher BMI (*p* < 0.005). The fetal birthweight was significantly higher with higher maternal BMI (*p* < 0.050). There were no other differences regarding obstetrical characteristics between the BMI groups (Table 1).

**TABLE 1:** Maternal, obstetrical characteristics and data related to the perineal laceration, degree, and repair, in relation to maternal body mass index (BMI).

		BMI < 18.5 (*N* = 203)	BMI 18.5–24.9 (*N* = 3984)	BMI 25.0–29.9 (*N* = 1662)	BMI 30–34.9 (*N* = 536)	≥35 (*N* = 210)	*p* value
Age, mean year, mean [SD]		28.4 [4.75]	29.74 [4.45]	29.24 [4.67]	29.09 [4.66]	28.60 [4.54]	0.050
Diabetes mellitus, *n* (%)	Yes	3 (1.5)	25 (0.6)	23 (1.4)	7 (1.3)	3 (1.4)	0.020
Inflammatory bowel disease, *n* (%)	Yes	3 (1.5)	37 (0.9)	9 (0.5)	1 (0.2)	2 (1.0)	0.127
Pre‐pregnancy dyspareunia (%^a^)	Yes	14 (6.9)	331 (8.3)	115 (6.9)	25 (4.7)	11 (5.2)	0.036
Pre‐pregnancy urinary incontinence, *n* (%^a^)	Yes	1 (0.5)	30 (0.2)	20 (1.2)	6 (1.1)	9 (4.2)	<0.005
Pre‐pregnancy stress urinary incontinence, *n* (%^a^)	Yes	0 (0.0)	8 (0.2)	4 (0.2)	2 (0.4)	4 (1.9)	0.306
Pre‐pregnancy urge urinary incontinence, *n* (%^a^)	Yes	1 (0.5)	7 (0.2)	5 (0.3)	2 (0.4)	2 (1.0)	0.432
Pre‐pregnancy gas and/or fecal incontinence, *n* (%^a^)	Yes	4 (2.2)	146 (3.7)	42 (2.5)	13 (2.4)	9 (4.2)	<0.005
Duration of second stage of labor in minutes, median [IQR]		43 [28–62]	40 [25–60]	38 [22–58]	33 [21–53]	29 [19–52]	<0.005
Fetal presentation at birth, *n* (%)	Occiput anterior	189 (93.1)	3754 (94.2)	1545 (93.0)	505 (94.2)	197 (93.8)	0.709
	Occiput posterior	12 (5.9)	176 (4.4)	91 (5.5)	24 (4.5)	7 (3.3)	
	Breech or foot	0	12 (0.3)	2 (0.1)	1 (0.2)	1 (0.5)	
	Other	2 (1.0)	38 (1.0)	22 (1.3)	6 (1.1)	3 (1.4)	
Episiotomy, *n* (%)	Yes	35 (17.2)	475 (11.9)	212 (12.8)	60 (11.2)	17 (8.1)	0.046
Mode of delivery, *n* (%)	Spontaneous vaginal	149 (73.4)	2890 (72.5)	1191 (71.7)	394 (73.5)	162 (77.1)	0.524
	Instrumental vaginal	54 (26.6)	1094 (27.5)	471 (28.3)	142 (26.5)	48 (22.9)	
Fetal birth weight, mean grams, [SD]		3514	3664	3721	3755	3786	0.050

^a^ The percentage was calculated with the number of respondents to the pre‐pregnancy questionnaire in the denominator.

Data related to the perineal laceration repair are presented in Table 2. The extent of the perineal laceration, degree III or IV, did not differ within the respective BMI group. The use of prophylactic antibiotics during the repair, suture techniques, and suture materials did not differ between BMI groups.

**TABLE 2:** Data related to the perineal laceration, degree, and repair, in relation to maternal BMI (body mass index).

		BMI < 18.5 (*N* = 203)	BMI 18.5–24.9 (*N* = 3984)	BMI 25–29.9 (*N* = 1662)	BMI 30–34.9 (*N* = 536)	BMI ≥ 35 (*N* = 210)	*p* value
Perineal laceration, *n* (%)	III‐degree	185 (91.1)	3667 (92.0)	1538 (92.5)	495 (92.4)	191 (91.0)	0.859
	IV‐degree	18 (8.9)	317 (8.0)	124 (7.5)	41 (7.6)	19 (9.0)	
Epidural anesthesia during surgery, *n* (%)	Yes	38 (18.7)	716 (18.0)	335 (20.2)	100 (18.7)	35 (16.7)	0.385
Prophylactic antibiotics during suturing, *n* (%)	Yes	84 (41.4)	1545 (38.8)	649 (39.0)	192 (35.8)	80 (38.1)	0.867
Suture technique external sphincter, 3d degree perineal laceration, *n* (%)	End‐ to‐ end	137 (67.5)	2966 (74.4)	1250 (75.2)	405 (75.6)	151 (71.9)	0.061
	Overlap	31 (15.3)	388 (9.7)	166 (10.0)	50 (9.3)	27 (12.9)	
Suture technique external sphincter, 4th degree perineal laceration, *n* (%)	End‐to‐end	13 (6.4)	236 (5.9)	88 (5.3)	30 (5.6)	10 (4.8)	0.768
	Overlap	4 (2.0)	61 (1.5)	25 (1.5)	7 (1.3)	5 (2.4)	

The 12‐month follow‐up questionnaires had a response rate of 52% (3457/6595 women). Outcomes analyzed with univariate logistic regression analyses are presented in Table 3.

**TABLE 3:** Patient‐reported complications after OASI from the one‐year follow‐up questionnaire.

	BMI ≤ 24.9		BMI 25.0–29.9			BMI ≥ 30		
	*N* (%)	OR	*N* (%)	OR	CI = 95%	*N* (%)	OR	CI = 95%
Reported to have had intercourse in last 3 months	1757/2124 (82.7)	1.00	695/829 (83.8%)	0.92	0.74–1.15	283/330 (85.8%)	0.80	0.57–1.10
Reported dyspareunia	925/2235 (41.4)	1.00	334/868 (38.5)	0.87	0.75–1.04	119/354 (33.6)	**0.72**	0.57–0.91
Reported urinary incontinence	217/2154 (10.1)	1.00	122/841 (14.5)	**1.51**	1.19–1.92	73/340 (21.5)	**2.44**	1.81–3.28
Reported stress incontinence	204/498 (41.0)	1.00	112/242 (46.3)	1.23	0.91–1.69	68/134 (50.7)	**1.49**	1.01–2.18
Reported urge incontinence	102/498 (20.5)	1.00	48/242 (19.8)	0.96	0.66–1.41	40/133 (30.1)	**1.67**	1.09–2.57
Reported sense of tight vagina during intercourse	468/2235 (20.9)	1.00	156/868 (18.0)	0.83	0.68–1.01	61/354 (17.2)	0.79	0.59–1.06
Reported sense of wide vagina during intercourse	132/2235 (5.9)	1.00	56/868 (6.5)	1.10	0.80–1.52	15/354 (4.2)	0.71	0.41–1.22
Reported sense of bulging	264/2140 (12.3)	1.00	107/836 (12.8)	1.04	0.82–1.33	34/331 (10.3)	0.81	0.56–1.19
Reported problems with emptying bowel	441/2148 (20.5)	1.00	164/838 (19.6)	0.94	0.77–1.15	85/339 (25.1)	1.23	0.99–1.69
Reported incontinence for gas or feces	742/2235 (33.2)	1.00	290/868 (33.4)	1.01	0.86–1.19	103/354 (29.1)	0.83	0.65–1.06
Reported gas incontinence^a^	526/739 (71.2)	1.00	218/290 (75.2)	1.23	0.90–1.67	72/102 (70.6)	0.97	0.62–1.53
Reported incontinence for loose stool^a^	121/740 (16.4)	1.00	44/289 (15.2)	0.92	0.63–1.34	27/103 (26.2)	**1.82**	1.12–2.94
Reported incontinence for solid stool^a^	52/739 (7.0)	1.00	21/289 (7.3)	1.04	0.61–1.75	4/103 (3.9)	0.53	0.19–1.51
Reported usage of pads for fecal incontinence^a^	48/741 (6.5)	1.00	14/290 (4.8)	0.73	0.40–1.35	5/103 (4.9)	0.74	0.29–1.90
Change of lifestyle due to fecal incontinence^a^	174/739 (23.5)	1.00	67/290 (23.1)	0.98	0.71–1.35	33/103 (32.0)	1.53	0.98–2.40
Wexner score ≥ 2	637/2235 (28.5)	1.00	250/868 (28.8)	1.02	0.85–1.21	99/354 (28.0)	0.97	0.76–1.25

*Note:* Bold values indicate a statistically significant association.

Abbreviations: BMI, body mass index; CI, confidence interval; OR, odds ratio.

^a^ The questions included in the Wexner score system.

Dyspareunia was significantly less common among overweight and obese women compared with normal‐weight women. The absolute rate of dyspareunia was 41.4% (*N* = 925) among normal‐weight women, 38.5% (*N* = 334) among overweight women, and 33.6% (*N* = 119) in the obese group. When adjusted for pre‐pregnancy dyspareunia, the risk of dyspareunia remained significantly lower among overweight women (adjusted OR = 0.82, CI = 0.68–0.98) with an even lower risk among obese women (adjusted OR = 0.71, CI = 0.54–0.93) as presented in Table 4. There was no significant difference over BMI strata concerning reported having had intercourse in the last three months, where the absolute rate for this variable, reporting having had intercourse the last three months, was 82.7% (*N* = 1757) among normal‐weight women, 83.8% (*N* = 695) and 85.8% (*N* = 283) among obese women. Furthermore, there was no significant difference in the reported sense of a tight or wide vagina between overweight and obese women compared with normal‐weight women.

**TABLE 4:** Adjusted patient‐reported complications after OASI according to BMI from the one‐year follow‐up questionnaire.

	BMI ≤ 24.9		BMI 25.0–29.9			BMI ≥ 30		
	*N* (%)	aOR	*N* (%)	aOR	CI = 95%	*N* (%)	aOR	CI = 95%
Reported dyspareunia^a^	925/2235 (41.4)	1.00	334/868 (38.5)	**0.82**	0.68–0.98	119/354 (33.6)	**0.71**	0.54–0.93
Reported urinary incontinence^b^	217/2235 (9.7)	1.00	122/868 (14.1)	**1.51**	1.11–2.04	73/354 (20.6)	**2.82**	1.94–4.12
Reported incontinence for gas or feces^c^	742/2235 (33.2)	1.00	290/868 (33.4)	0.98	0.76–1.27	103/354 (29.1)	1.22	0.84–1.77

*Note:* Bold values indicate a statistically significant association.

Abbreviations: aOR, adjusted odds ratio; BMI, body mass index; CI, confidence interval.

^a^ Adjusted for pre‐pregnancy dyspareunia.

^b^ Adjustments were made for age, pre‐pregnancy urinary incontinence, and fetal birth weight.

^c^ Adjusted for age, inflammatory bowel disease (IBD), pre‐pregnancy gas and/or feces incontinence, fetal birth weight, prophylactic antibiotic during repair, suture technique of grade 3 perineal laceration.

There was an increased risk of UI of 51% among overweight women when adjusted for age, pre‐pregnancy urinary incontinence and fetal birthweight (adjusted OR = 1.51, CI = 1.11–2.04) compared with normal‐weight women, as seen in Table 4. The corresponding adjusted OR for obese women showed a nearly threefold risk of UI (aOR = 2.82, CI = 1.94–4.12). SUI was not significantly more common among overweight women (OR = 1.24, CI = 0.91–1.70), compared with normal‐weight women, whereas obese women had a significantly higher risk of SUI (OR = 1.49, CI = 1.01–2.18), compared with normal‐weight women.

The risk of UUI was not higher among overweight women (OR = 0.96, CI = 0.66–1.41) compared with normal‐weight women, while obese women showed a significantly higher risk (OR = 1.67, CI = 1.09–2.57) compared with normal‐weight women for UUI.

The absolute rate of any reported anal incontinence was 33.2% (*N* = 742) among normal‐weight women, 33.4% (*N* = 290) among overweight women, and 29.1% (*N* = 103) in the obese group. Risk estimates for any reported gas and/or feces incontinence, with adjustments for age, inflammatory bowel disease, pre‐pregnancy gas and/or feces incontinence, fetal birthweight, prophylactic antibiotic during repair, and suture technique grade 3, did not show any difference among overweight and obese women compared with women with a BMI ≤ 24.9 (Table 4). There was a significantly higher risk of leakage of loose stool among obese women (OR = 1.82, CI = 1.12–2.94) compared with normal‐weight women, but none of the other Wexner score variables were influenced by BMI. Furthermore, the effect on quality of life based on total Wexner score, with a cut‐off level of a total Wexner score ≥2^10^ was not affected by maternal BMI. The rate of reporting a sense of vaginal bulging and the need of splinting during defecation was somewhat lower among overweight and obese women compared with normal‐weight women, but the association was not statistically significant.

## 4 DISCUSSION

In women with a first‐time vaginal birth complicated by an OASI, the rate of dyspareunia—pain during intercourse—was 39% at 12 months postpartum. There was a significant difference with lower prevalence in overweight and obese women compared with normal‐weight women, even when adjusted for prepregnancy dyspareunia rates. Other symptoms associated with dyspareunia, such as a sensation of a tight or wide vagina during intercourse, did not differ between the BMI groups. This finding has not been described before.

This study is based on self‐reported symptoms 12 months postpartum and among women with a first‐time vaginal birth complicated by an OASI. The self‐reported rate of having had intercourse in the last 3 months did not differ between the BMI groups, which is of value in the assessment of differences in self‐reported symptoms of dyspareunia and related symptoms.

An increased risk of postpartum dyspareunia after an OASI is related to both obstetric events and maternal characteristics. Higher maternal age and smoking have been shown to be more common among women with dyspareunia 12 months postpartum.^23^ Among older women, overweight and obesity seem to influence most aspects of sexual function, including pain, negatively.^24^ A study conducted by Gommesen et al. showed that women with a higher degree of perineal laceration reported more symptoms of dyspareunia 12 months postpartum. In their study, there was a tendency for dyspareunia to be less common among obese women compared with normal‐weight women, regardless of the degree of perineal laceration, although it did not reach statistical significance.^23^ Congruent findings regarding dyspareunia and BMI during pregnancy have been found in a cross‐sectional study by Ribeiro et al. They assessed sexual function, using the Female Sexual Function Index (FSFI) during pregnancy, where the results of the study showed a decreased risk of dyspareunia in the third trimester of pregnancy in obese women compared with normal‐weight women, but without any postpartum data.^25^

There was an increased risk of UI among overweight and obese women compared with normal‐weight women at 12 months after a vaginal birth with OASI, and further an increased risk of SUI at this point. This finding is congruent with a previous study on women with OASI eight weeks postpartum, where the risk of UI was increased in overweight and obese women.^18^ Traditionally, this finding has been explained by the strain of the weight on the pelvic floor, but with an increasing understanding of the function of the pelvic floor muscles and especially the levator ani muscle, a tangible muscle injury implies a weakened function of the whole pelvic floor. A recent study by Volløyhaug et al. showed that 29.4% of women with OASI also had a major injury of the levator ani muscle.^26^

Regarding anal incontinence, our study did not show any differences in the prevalence of self‐reported symptoms over the BMI strata. In contrast to our result, a prospective study showed that women with obesity had a higher risk of AI at 12 months after an OASI compared with normal‐weight women.^27^ Gommesen et al. had a smaller study population than in the present study and used a different scoring system to detect AI, which might explain the different findings.^27^

The overall prevalence of AI and UI after an OASI varies in the literature, as do the risk factors and used definitions of AI and UI in primiparous women.^11^, ^12^, ^13^, ^28^, ^29^ Different follow‐up questionnaires, timing, methods of follow‐up questions, and definitions of incontinence might account for these differences.^5^, ^13^ Previous studies imply the need for individualized counseling about PFD and dyspareunia postpartum.^23^, ^30^, ^31^, ^32^ The finding that obese and overweight women had a decreased risk of postpartum dyspareunia, no difference in the risk of AI, and an increased risk of overall UI and stress urinary incontinence (SUI) 12 months after sustaining an OASI compared with normal‐weight women is of great value to facilitate individualized counseling postpartum after an OASI.^9^, ^33^ Obesity itself is a risk factor for not getting adequate healthcare due to weight bias in healthcare.^34^ This should be taken into account in clinical counseling.

A strength of this study is that it is population‐based and contains self‐reported symptoms. The PLR contains data on a sufficiently large population to make it possible to analyze subgroups. Furthermore, it is a strength that data is registered from several centers throughout Sweden, which makes the results generalizable to similar settings. Despite a response rate of 52%, the study included a large sample of women across multiple clinics, which supports the robustness of the findings. Nonetheless, caution is warranted when generalizing the results to all postpartum women.

In this large reference group of women with a first‐time vaginal delivery, complicated by an OASI, the follow‐up was carried out using collected data from questionnaires on self‐reported symptoms. This might be considered a limitation as it was not followed by a clinical examination, but it mirrors the impact of the symptoms that PFD have on everyday activities. Recent studies have shown the importance of considering subjectively reported problems postpartum, independent of anatomic appearance, as these symptoms often cause feelings of shame and embarrassment.^21^, ^35^ This study has several limitations that should be acknowledged. A limitation of the study is that at the time of data extraction, the Perineal Tear Register included data from approximately 85% of all delivery clinics in Sweden. Although nationwide coverage was not yet complete, the available data from the study period include a large study population. Future studies using more recent data with complete nationwide coverage may allow inclusion of an even larger study population.

A potential limitation of this study is recall bias, as pre‐pregnancy information was collected postpartum, as women could be enrolled only after sustaining a perineal tear. However, as the analyses focused on comparisons between BMI groups, there is no reason to assume that recall of the pre‐pregnancy symptoms differed systematically by BMI category, and any recall bias is therefore likely to be non‐differential and to affect all BMI groups similarly.

Further limitations of this register‐based study are that except for the Wexner score, the questions are only a few among those in completely validated questionnaires about pelvic floor dysfunction. For the unique PLR general public use, questionnaire brevity is of essence as to not put women off the task of responding.^36^ All questionnaires have been tested in focus groups of women to ensure a reasonable balance between too many and too few questions asked.

The lack of data on breastfeeding and use of contraceptives in the present study is a limitation. Both breastfeeding and the use of contraceptives have been shown to have an impact on dyspareunia postpartum, regardless of perineal laceration.^31^, ^37^ Furthermore, previous studies have shown a lower incidence of breastfeeding among obese women,^38^, ^39^ which could be another factor contributing to the lower risk of dyspareunia in this group, though this was not investigated in this study.

## 5 CONCLUSION

Dyspareunia 12 months after a first‐time vaginal birth complicated by an OASI is, although common, reported less frequent among overweight and obese women than among normal‐weight women. Self‐reported symptoms of AI do not differ between the BMI groups, while the risk of UI increased with higher BMI. The new finding of a lower risk of dyspareunia with higher BMI raises the hypothesis that adipose tissue in the pelvic floor may protect against dyspareunia.

## AUTHOR CONTRIBUTIONS

**Linda Hjertberg**: Conceptualization; methodology; formal analysis; investigation; data curation; writing original draft. **Eva Uustal**: Conceptualization; methodology; validation; writing review and editing. **Marie Blomberg**: Conceptualization; methodology; supervision; project administration; funding acquisition. **Sofia Pihl**: Conceptualization; methodology; writing review and editing; visualization. All authors gave final approval and agreed to be accountable for all aspects of work, ensuring integrity and accuracy.

## FUNDING INFORMATION

Financial support was received from the County Council of Östergötland and Linköping University, Sweden (ALF grants, Region Östergötland).

## CONFLICT OF INTEREST STATEMENT

None of the authors have any conflicts of interest to declare.

## ETHICS STATEMENT

The Regional Ethical Review Board in Linköping approved the study on April 20, 2016 (Dnr 2016/144–31).

## Supporting information


**Appendix S1.** Pre‐pregnancy questionnaire.

**Appendix S2.** Questionnaire one‐year follow‐up after an OASI.

## Data Availability

The datasets generated and/or analyzed during the current study are not publicly available due to restrictions in Swedish law (Offentlighets‐och sekretesslag [SFS 2009:400]/Public Access to Information and Secrecy Act [SFS 2009:400]) but are available from the corresponding author on reasonable request.
