# Supplementary material for: Self‐reported pelvic floor dysfunction 12 months after an obstetric anal sphincter injury in relation to maternal body mass index
Source: Acta Obstet Gynecol Scand. 2026 Mar 5;105(7):1200–9. doi: 10.1111/aogs.70171 (PMC13308988; doi:10.1111/aogs.70171)
Supplement: Supplementary file 2 — Appendix S2. Questionnaire one‐year follow‐up after an OASI. [file AOGS-105-1200-s001.pdf]

Dear

You had surgery/received treatment at our clinic 1 year ago.

DO NOT COPY

Your assessment after surgery
(about 1 year)

Personal identity number:<br>Name<br>Address<br>Postal code City<br>Phone<br>Email

⇦ ***If the above information is missing or incorrect, please fill in here***

Your questionnaire can also be found online. It is easier for us if you complete the questionnaire online.
Go to www.gynop.se, click "LOG IN".
Your password is

There are gaps in the numbering of questions since certain questions are not relevant for you.

1. Date when the questionnaire is completed: ................-.........-........

2. Do you have pain in the pelvic area/lower abdomen?

☐ No

☐ Yes Specify the severity of your pain

- Menstrual cramps, regular monthly cramps
  The pain lasts between .... and .... days.
  ☐ No, no pain
  ☐ Yes, mild pain
  ☐ Yes, moderate pain
  ☐ Yes, severe pain
  ☐ Yes, unbearable pain

- Abdominal pain
  ☐ No, no pain
  ☐ Yes, mild pain
  ☐ Yes, moderate pain
  ☐ Yes, severe pain
  ☐ Yes, unbearable pain

- Pelvic pain, describe: ....
  ....
  ....
  ☐ No, no pain
  ☐ Yes, mild pain
  ☐ Yes, moderate pain
  ☐ Yes, severe pain
  ☐ Yes, unbearable pain

DO NOT COPY

3a. Have you had menstrual periods/vaginal bleeding during the past year? ☐ No ☐ Yes

3b. Are your menstrual periods regular? ☐ Yes ☐ No

3c. Do you experience spotting/unexpected vaginal bleeding? ☐ Yes ☐ No

3d. Do you take hormones that regulate your period? ☐ Yes ☐ No

3e. How would you describe this bleeding?

☐ No menstruation/cessation of menstruation
☐ Light
☐ Moderate
☐ Heavy
☐ Very heavy
☐ Varies from time to time

4. Do you have menopausal symptoms (hot flushes, sweating, palpitations)?

☐ No
☐ Yes
☐ Don't know

5. Do you take hormones containing oestrogen?

☐ No
☐ Yes, for menopausal symptoms
☐ Yes, for pelvic problems
☐ Yes, for problems with urine/urinary tract
☐ Yes, for another reason ....

6a. Do you have a feeling that something is bulging out from the vagina?

☐ Never ☐ Almost never ☐ 1–3 times per month ☐ 1–3 times per week ☐ Daily

6b. Do you experience chafing in the genital area?

☐ Never ☐ Almost never ☐ 1–3 times per month ☐ 1–3 times per week ☐ Daily

6c. Do you use a pessary to prevent prolapse?

☐ No, have never had a pessary
☐ No, not now, but have previously had a pessary
☐ Yes, I have a pessary now
☐ Don't know

DO NOT COPY

7a. Do you have problems emptying your bladder?

☐ Never ☐ Almost never ☐ 1–3 times per month ☐ 1–3 times per week ☐ Daily

7b. Have you had problems with urinary urgency (sudden onset of a strong need to urinate)?

☐ Never ☐ Almost never ☐ 1–3 times per month ☐ 1–3 times per week ☐ Daily

7c. Do you need to get up at night to urinate?

☐ Never ☐ Almost never ☐ Usually one time ☐ Usually two times ☐ More than two times

7d. Do you experience urinary leakage or involuntary urination?

☐ Never ☐ Almost never ☐ 1–3 times per month ☐ 1–3 times per week ☐ Daily

If you answered Never – Almost never to question 7d above, go to question 9a

8a. Do you experience leakage of urine when you get up out of bed? ☐ Yes ☐ No

8b. How often do you experience leakage of urine associated with physical activity, or when you laugh, cough or sneeze?

☐ Never
☐ 1–4 times per month
☐ 1–6 times per week
☐ Once a day
☐ More than once a day

8c. How much urine usually leaks with physical activity, or when you laugh, cough or sneeze? *(The question is asked so that we can estimate the quantity of urine leaked).*

☐ No leakage
☐ Damp underwear
☐ Wet underwear
☐ Soaks through clothing
☐ Runs down legs or onto floor

DO NOT COPY

8d. How often do you experience a sudden onset of a strong need to urinate, and leak urine before you reach the toilet?

- ☐ Never
- ☐ 1–4 times per month
- ☐ 1–6 times per week
- ☐ Once a day
- ☐ More than once a day

8e. How much urine usually leaks when you have urinary urgency (sudden onset of a strong need to urinate)?*(The question is asked so that we can estimate the quantity of urine leaked).*

- ☐ No leakage
- ☐ Damp underwear
- ☐ Wet underwear
- ☐ Soaks through clothing
- ☐ Runs down legs or onto floor

8f. Does urine leak both with physical activity (e.g. cough, heavy lifting, exercise) and with urinary urgency (sudden onset of a strong need to urinate)? ☐ Yes ☐ No

8g. If yes, which one is most problematic?
- ☐ Leakage with physical activity causes greater discomfort than leakage with urgency
- ☐ Leakage with urgency causes greater discomfort than leakage with physical activity
- ☐ Equal discomfort from leakage with urgency and leakage with physical activity

8h. Do you avoid activities (e.g. physical exercise or going out) because you are afraid of leakage?

☐ Never ☐Seldom ☐Occasionally ☐Frequently ☐Always

8i. Do you avoid places and situations where you know it is difficult to find a toilet?

☐ Never ☐Seldom ☐Occasionally ☐Frequently ☐Always

Mark an X next to yes or no **for each question at 8j**

8j. Does your urinary leakage affect

your holidays?	☐ Yes	☐ No
your family life?	☐ Yes	☐ No
your sex life?	☐ Yes	☐ No
your social life (going out, meeting friends, etc.)?	☐ Yes	☐ No
your sleep at night?	☐ Yes	☐ No
your working life?	☐ Yes	☐ No

DO NOT COPY

9a. Do you ever have problems emptying your bowels?

☐ Never ☐ Almost never ☐ 1–3 times per month ☐ 1–3 times per week ☐ Daily

9b. Do you ever have to push against the back wall of the vagina to empty your bowels?

☐ Never ☐ Almost never ☐ 1–3 times per month ☐ 1–3 times per week ☐ Daily

10a. Do you have problems holding in stool or gas?

☐ No

☐ Yes

If you answered no to the above question, skip to question 11a

10b. Do you ever pass gas even when it is inappropriate?

☐ Never

☐ Almost never

☐ Yes, 1-3 times a month

☐ Yes, 1-3 times a week

☐ Yes, daily

10c. Do you experience leakage of loose stool?

☐ Never

☐ Almost never

☐ Yes, 1-3 times a month

☐ Yes, 1-3 times a week

☐ Yes, daily

10d. Do you experience leakage of firm stool?

☐ Never

☐ Almost never

☐ Yes, 1-3 times a month

☐ Yes, 1-3 times a week

☐ Yes, daily

DO NOT COPY

10e. Do you use sanitary pads/protection because of stool leakage?

☐ Never

☐ Almost never

☐ Yes, 1-3 times a month

☐ Yes, 1-3 times a week

☐ Yes, daily

10f. Does your leakage problem affect your lifestyle?

☐ Never

☐ Almost never

☐ Yes, 1-3 times a month

☐ Yes, 1-3 times a week

☐ Yes, daily

12a. Have you had intercourse during the past 3 months?

☐ Yes ☐ No ☐ Not applicable ☐ Prefer not to answer

12b. If yes on question 15a, do you experience pain in the genital area during intercourse?

☐ No, no pain

☐ Yes, mild pain

☐ Yes, moderate pain

☐ Yes, severe pain

☐ Yes, unbearable pain

12c. If yes on question 12a, do you experience leakage of urine during intercourse?

☐ Yes

☐ No

12d. If yes on question 12a, do you feel

that your vaginal opening is too small/narrow?	☐ Yes ☐ No
that your vaginal opening is too large/open?	☐ Yes ☐ No
pain in the vaginal opening?	☐ Yes ☐ No
other symptoms from the vaginal opening?	☐ Yes ☐ No

If yes, what type of symptoms? …………………………………………………………….

……………………………………………………………………………………………

DO NOT COPY

13. How do you feel about the surgical outcome so far? My condition is:

☐ Greatly improved
☐ Improved
☐ Unchanged
☐ Worse
☐ Much worse

14. During the time two months after surgery until now, have you had new problems/complications related to your surgery?

☐ No, skip to question 22
☐ Yes, mild
☐ Yes, severe/serious

15. Are these problems/complications serious enough to be reported? (e.g. to patient insurance)

☐ No ☐ Yes

16a. During the time two months after surgery until now, have you had to seek medical care because of these problems/complications?

☐ No ☐ Yes

**If yes**, what medical facility did you visit?	Name of department and hospital/primary care centre that you visited
☐ The department where you had surgery<br><br>☐ Primary care centre<br><br>☐ Other medical facility	 <br><br>...<br><br>...

16b. If you answered yes to question 16a: Were you hospitalised because of the complication?

☐ No, I left the hospital the same day.
☐ Yes, stayed for one night.
☐ Yes, stayed for two or more nights.

DO NOT COPY

16c. If you answered yes to question 16a: Did treatment of the complication include surgery?

☐ No ☐ Yes

16d. If you answered yes to question 16a: Did these problems/complications lead to any other intervention?

☐ No
☐ Yes, monitored with additional recheck and/or investigation
☐ Yes, pain relieving medicine
☐ Yes, other treatment. Describe: ....
....

16e. If you answered yes to question 11a: Did the complication cause your sick leave to be extended?

☐ No
☐ Yes, number of weeks ………………..

Describe your problems/complications by choosing one or more of the following options.

17a. What organ(s) was/were affected?

☐ Abdominal scar, surgical scar

☐ Nerve/Sensation

☐ Uterus

☐ Urinary bladder

☐ Ureter from the kidney to the urinary bladder

☐ Urethra

☐ Vagina

☐ Intestines

☐ Other (Describe in question 17c)

DO NOT COPY

17b Different types of problems/complications. Mark with an X if you have had any of the following:

- ☐ Pain with urination for more than 2 months following surgery
- ☐ Urinary retention that required a medical visit, urinary drainage
- ☐ Urinary leakage/urinary incontinence
- ☐ Hernia in the surgical scar (incisional hernia)
- ☐ Prolapse
- ☐ Bowel obstruction (ileus/subileus)
- ☐ Abnormal connection to the vagina, intestine or urinary bladder (fistula)
- ☐ Groin pain
- ☐ Pain at the pubic bone
- ☐ Pain in abdominal wall
- ☐ Other (Describe in question 17c)

17c. Describe the problems/complications you have experienced: ....

....

....

....

....

18. Do you still have problems related to the operation?

☐ No
☐ Yes If yes, describe: ....

19. If you answered yes to any of questions 14-18, would you consent to allow us to read the relevant medical records before your surgery?

☐ No
☐ Yes

20. How do you feel about the results following surgery?

☐ Very satisfied
☐ Satisfied
☐ Neither satisfied nor dissatisfied
☐ Dissatisfied
☐ Very dissatisfied

DO NOT COPY

24. Have you had any problems understanding any question(s) in the questionnaire?

☐ No ☐ Yes

If Yes, write the number of the question and describe the problem:

....

....

....

....

....
Name (of the person who filled in the questionnaire)

DO NOT COPY
